# The Enhanced Musical Rhythmic Perception in Second Language Learners

**DOI:** 10.3389/fnhum.2016.00288

**Published:** 2016-06-10

**Authors:** M. Paula Roncaglia-Denissen, Drikus A. Roor, Ao Chen, Makiko Sadakata

**Affiliations:** ^1^Institute for Logic, Language and Computation, University of AmsterdamAmsterdam, Netherlands; ^2^Amsterdam Brain and Cognition (ABC), University of AmsterdamAmsterdam, Netherlands; ^3^Musicology Department, University of AmsterdamAmsterdam, Netherlands; ^4^Utrecht Institute of Linguistics (Uil OTS), Utrecht UniversityUtrecht, Netherlands; ^5^Donders Institute for Brain, Cognition and Behavior, Radboud University NijmegenNijmegen, Netherlands

**Keywords:** music rhythm, speech rhythm, second language

## Abstract

Previous research suggests that mastering languages with distinct rather than similar rhythmic properties enhances musical rhythmic perception. This study investigates whether learning a second language (L2) contributes to enhanced musical rhythmic perception in general, regardless of first and second languages rhythmic properties. Additionally, we investigated whether this perceptual enhancement could be alternatively explained by exposure to musical rhythmic complexity, such as the use of compound meter in Turkish music. Finally, it investigates if an enhancement of musical rhythmic perception could be observed among L2 learners whose first language relies heavily on pitch information, as is the case with tonal languages. Therefore, we tested Turkish, Dutch and Mandarin L2 learners of English and Turkish monolinguals on their musical rhythmic perception. Participants’ phonological and working memory capacities, melodic aptitude, years of formal musical training and daily exposure to music were assessed to account for cultural and individual differences which could impact their rhythmic ability. Our results suggest that mastering a L2 rather than exposure to musical rhythmic complexity could explain individuals’ enhanced musical rhythmic perception. An even stronger enhancement of musical rhythmic perception was observed for L2 learners whose first and second languages differ regarding their rhythmic properties, as enhanced performance of Turkish in comparison with Dutch L2 learners of English seem to suggest. Such a stronger enhancement of rhythmic perception seems to be found even among L2 learners whose first language relies heavily on pitch information, as the performance of Mandarin L2 learners of English indicates. Our findings provide further support for a cognitive transfer between the language and music domain.

## Introduction

Language and music have many features in common which could suggest a common origin (Wallin et al., 2001; Mithen, 2005). Different views on their origin have proposed that either music might be a byproduct of language (Pinker, 1997), language could have originated from music (e.g., Darwin, 1872; Falk, 2004; Fitch, 2010) or language and music could have originated from a common cognitive domain (Brown, 2000). Despite these different views, investigating possible shared features of these domains might shed more light on what could be a human innate ability, indicate an evolutionary adaptation, or be a byproduct of the other domain. Thus, investigating a possible common feature in language and music might provide the necessary tools to better understand their origin and the evolution of these features in the cognitive landscape (Patel, 2006).

The use of a common mechanism in language and music has been suggested in syntactic processing (Patel, 1998, 2003a, 2008) as well as in melodic and rhythmic organization (Lerdahl and Jackendoff, 1983; Jackendoff, 1989). Evidence of such commonalities has been provided by studies reporting a transfer effect of expertise between these two domains. On the one hand, sensitivity to pitch processing in language appears to be transferred to the music domain (Deutsch et al., 2006, 2009; Elmer et al., 2011). On the other hand, melodic aptitude positively correlates with pronunciation skills in second language (L2; Milovanov et al., 2008) and phonological perception (Slevc and Miyake, 2006; Marques et al., 2007). Furthermore, it has been suggested that musical training improves first-language reading and syntactic skills (Jentschke and Koelsch, 2009; Moreno et al., 2009; Brod and Opitz, 2012; Tierney and Kraus, 2013), while musical rhythmic training, more specifically, improves reading impairments (Bhide et al., 2013).

In both language and music, rhythm is used to organize the sound stream, grouping acoustic events such as sounds and pauses into meaningful units, e.g., words and sentences in language, and phrase and motive in music. While linguistic rhythm helps speech comprehension (Roncaglia-Denissen et al., 2013b) and creates acoustically marked boundaries inside and between words (Patel, 2003b), musical rhythm generates temporal expectation at different hierarchical levels (Patel, 2006). Perhaps, in the same way that musical training may shape one’s auditory perception (cf. Vuust et al., 2012), learning a second language (L2) could enhance the perception of rhythmic variation in language, such as sound duration and intensity, which are also present in music organization. Therefore, a rhythmic perceptual enhancement could be created in the music domain via language.

In previous research, Roncaglia-Denissen et al. (2013a) reported that mastering languages with different rhythmic properties, e.g., German and Turkish, helps to enhance individuals’ musical rhythmic perception. The authors argued that the rhythmic differences between the two languages could account for this enhancement: German is a stress-timed language and uses the metric foot as its unit of speech organization, i.e., a combination of one stressed syllable with at least one unstressed syllable (Nespor and Vogel, 1986). Turkish, on the other hand, is considered a syllable-timed language and uses syllable, regardless of stress, as its speech organization unit. In terms of word-level metrical stress, Turkish is by default word final (Inkelas and Orgun, 2003), while German is trochaic or word initial (Eisenberg, 1991)^1^.

The current research aims to further investigate the impact of learning a L2 on the musical rhythmic perception. Therefore, we tested one group of Turkish monolinguals and three groups of L2 learners (Dutch, Mandarin and Turkish L2 learners of English) on their ability to discriminate rhythmic variation in music. If learning a L2 helps to enhance one’s rhythmic perception, then all L2 learner groups should be better than the monolingual group at musical rhythmic perception.

Alternatively to the suggestion that L2 learning impacts musical rhythmic perception, it has been proposed that an enhanced musical rhythmic perception could result from the exposure to music complexity, such as the use of compound meter in Turkish music (Hannon et al., 2012). If this should be the case then all Turkish participants, both monolinguals and L2 learners of English, should be better at musical rhythmic perception than non-Turkish participants. No differences between Turkish monolinguals and Turkish L2 learners of English should be found.

By testing L2 learners of English from different native languages, the current research aims to investigate how rhythmic differences between first and second languages might affect one’s rhythmic perception. Regarding their rhythmic properties, Dutch and English are considered both stress-timed languages with the metric preference for the trochee, i.e., a stressed syllable followed by an unstressed one (Pike, 1945; Jusczyk et al., 1993; Vroomen and de Gelder, 1995). Therefore, Dutch L2 learners of English should have one single set of rhythmic properties as a result of the full rhythmic overlap between these two languages.

Turkish, on the other hand, is a syllable-timed language (Inkelas and Orgun, 2003; Van Kampen et al., 2008). At the word level, Turkish has a preference for word-final stress, thus, Turkish L2 learners of English could show an enhanced musical rhythmic perception as a result of encoding distinct sets of rhythmic properties of their first and second languages. This enhanced musical rhythmic perception could be reflected by better performance in musical rhythmic discrimination than Dutch L2 learners of English.

In Mandarin, which is also considered a syllable-timed language (Goswami et al., 2010), the importance of tonal variation for its lexical system is well established (Leather, 1983; Moore, 1993; Shen, 1993; Lai and Sereno, 2007). Thus, we expect Mandarin L2 learners of English to perform better in detecting melodic variation than their L2 learner peers. However, at the word level, there is no consensus regarding the lexical stress preference in Mandarin (Shen, 1993; Duanmu, 1999; Zhang et al., 2008), and lexical stress is restricted to a small percentage of words. That is, while the initial syllable carries a canonical lexical tone, a non-initial syllable carries a neutral tone, perceptually weaker in comparison to the canonical tone. The remaining non-neutral tone words cannot be categorized as either trochaic or iambic (Chao, 1968; Moore, 1993; Zhang et al., 2008).

Regarding the Mandarin compared to Turkish L2 learners of English, two hypotheses can be made. First, it could be that Mandarin L2 learners of English perform worse than Turkish L2 learners of English. This could be the case because the lexical stress system of Mandarin relies more on pitch than on the rhythmic information of sound duration and intensity, while the lexical stress system of English is mainly based on rhythmic features. Learning a feature in L2 that is not present in the first language might result in a negative transfer with a more effortful and less native-like outcome (Ullman, 2001, 2004). Therefore, the benefits from first and L2 rhythmic differences possible for Turkish L2 learners of English could be hindered in Mandarin natives.

Second, Mandarin L2 learners of English may perform comparably to Turkish L2 learners of English. This could be the case because both the establishment of a broader lexical stress system by Mandarin natives and the reconfiguration of the default stress position by the Turkish natives might require adjusting their rhythmic perception to accommodate it to their L2 as well. In this case, both groups could be sensitive to different rhythmic properties, such as sound duration and intensity, as a result of mastering two rhythmically distinct languages. Hence, both groups could show an enhanced perception of rhythmic variation and comparable performances in rhythmic discrimination.

Regardless of whether Mandarin L2 learners of English perceive musical rhythm comparably to or worse than Turkish L2 learners of English, the perception of musical rhythmic variation of Mandarin L2 learners of English should still be higher than of Dutch L2 learners of English. This should be the case because, even though secondary, the rhythmic features of sound intensity and duration are still used to some extent for stress perception in tonal languages (Shen, 1993; Lai and Sereno, 2007).

## Materials and Methods

### Participants

Sixty participants^2^, all non-musicians, were divided into four experimental groups, i.e., 15 Mandarin L2 learners of English (8 females, *M*_age_ = 25.06 years, *SD* = 1.98, mean age of L2 first exposure, AoL2FE = 9.93 years, *SD* = 2.31), 15 Turkish L2 learners of English (8 females, *M*_age_ = 26.33 years, *SD* = 3.08, *M*_AoL2FE_ = 10.13 years, *SD* = 4.34), 15 Dutch L2 learners of English (8 females, *M*_age_ = 25.53 years, *SD* = 4.64, *M*_AoL2FE_ = 8.80 years, *SD* = 3.27) and 15 Turkish monolinguals^3^ (8 females, *M*_age_ = 18.93 years, *SD* = 1.94). Participants reported having little formal musical training (*M* = 1.61 years, *SD* = 2.19) and were all university students or had recently graduated. None of the participants reported any neurological impairment or hearing deficit, and all had normal or corrected-to-normal vision. This study was approved by the ethics committees of the Faculty of Humanities of the University of Amsterdam, Utrecht University and the Middle East Technical University, in Ankara. All participants gave their written informed consent for data collection, use and publication.

### Materials

#### Phonological and Working Memory Measures

To measure participants’ phonological memory, i.e., the ability to store and recall novel sounds (cf., Baddeley et al., 1998), the Mottier test was used (Mottier, 1951). The Mottier test is a non-word repetition task composed of six sets of non-words, ranging from two to six syllables each. All non-words consisted of the constant syllabic structure of one consonant followed by one vowel, i.e., CV. For the Dutch participants, the stimulus material followed the Dutch phonetic rules and was spoken by a male native speaker. For the Turkish and Mandarin participants, the stimulus material was spoken by a female native speaker of each language according to the phonetic rules of Turkish and Mandarin, respectively.

Participants’ working memory capacity was measured using the backward digit span, a cognitive task involving information storage and transformation (Oberauer et al., 2000; Süß et al., 2002). The backward digit span version here used was composed of 14 sets of two trials, ranging from two to eight numbers. In the Dutch version, numbers were spoken by a male native speaker, while in the Mandarin and Turkish versions, by a female native speaker.

#### Melodic Aptitude Test

Melodic aptitude tests have been used by the field literature as an indicator of musical aptitude (Seashore et al., 1960; Gordon, 1965, 1969; Wallentin et al., 2010; Roncaglia-Denissen et al., 2013a). Participants’ melodic aptitude was assessed using the melodic subset of the musical ear test (MET; Wallentin et al., 2010). The melodic aptitude test consisted of 52 pairs of melodic phrases, presenting 3–8 tones. The melodies had the duration of one measure and were played at 100 bpm. Different trials (26 pairs) contained pitch violation and in half of them (13 pairs) the pitch violation was also a violation in the pitch contour. Twenty-five trials were constituted by non-diatonic tones, 20 were in the major keys and seven in minor keys. The order in which these features occurred was randomized.

#### Rhythmic Aptitude Test

The rhythmic subset of the MET (Wallentin et al., 2010) was used as a measure of musical rhythmic aptitude. The rhythmic subset comprised 52 pairs of rhythmic phrases that were either identical or different from each other. Rhythmic phrases were recorded using wood blocks and were 4–11 beats long. All phrases had the duration of one measure and were played at 100 bpm. Trials consisting of two distinct rhythmic phrases differed only with regard to one beat. Rhythmic complexity was achieved by including even beat subdivisions in 31 trials and triplets in the remaining 21 trials. Thirty-seven trials began on the downbeat and the remaining 15 trials started after it. The order in which these features occurred was randomized.

#### Self-Reported Language Skills and History Questionnaire

Participants were given a self-reported language skills and history questionnaire. Self-reported language skills have been shown to correlate highly with objective measures of language skills (Marian et al., 2007) and were successfully used in previous research to assess individuals’ language skills (e.g., Garbin et al., 2011; Roncaglia-Denissen et al., 2015). The language skills and history questionnaire in the current study is the same one used and published by Roncaglia-Denissen et al. (2013a) in previous study. In this questionnaire, participants’ first and second languages’ listening, writing, reading and speaking skills are assessed, together with their age of first and L2 first exposure, situations of acquisition, and current use. Based on the results of the assessment and on participants’ own perception of their language preference, English was regarded as the L2 in all L2 learners groups, and no group differences were found in terms of age of L2 first exposure, *p* = 0.45.

#### Music Background Questionnaire

Participants were given a music background questionnaire to assess information about their formal musical training (number of years) and daily exposure to music (hours). Formal musical training was assessed for each participant in terms of number of years they attended to music lessons to learn an instrument or to learn how to sing. Whether they learned one or multiple instruments at this period or whether an instrument was learned simultaneously with singing lessons was disregarded. The music background questionnaire is provided in the supplementary material.

### Procedures

Participants were tested individually in a quiet room^4^. The tests were administered in a different pseudo-randomized order for each participant and each individual session lasted approximately 40 min. For the rhythmic and melodic aptitude tests, participants performed two practice trials prior to each test which could be repeated until the test at hand was fully understood. Practice trials were not presented again and were not part of the experimental items. At the end of the session, participants were given a self-reported language skills and history questionnaire and a music background questionnaire.

#### Mottier Test, Backward Digit Span

In the Mottier test, participants heard non-words and were instructed to repeat each word as accurately as possible immediately after hearing it. Participants’ responses were computed *ad hoc* by the experimenter. The test was terminated when participants failed to recall a minimum of four items correctly in the same set. Participants’ scores were based on the total number of correctly recalled non-words, with a maximum score of 30 non-words.

In the backward digit span, participants listened to sequences of numbers while facing away from the computer. At the end of each trial, participants were instructed to repeat the numbers in the reversed order in which they were presented. The test was terminated when participants failed to correctly recall one trial of the same set. Participants’ scores were given based on the total number of trials correctly recalled with a maximum of 14 trials.

#### Melodic Aptitude Test

In the melodic aptitude test, participants were presented with the stimulus material via the computer. Mandarin L2 learners of English were given an answer sheet, marking down if the previously heard trial was composed by identical or non-identical melodic phrases. The remaining participants performed this test via the computer and their responses were collected by pressing the corresponding answer-key on a computer key-board. The position of the correct-response key was counter-balanced across participants.

#### Rhythmic Aptitude Test

Participants were presented with rhythmic pairs containing either identical or different rhythmic phrases. At the end of each trial, participants had to decide if the rhythmic phrases of the same trial were identical or not. Mandarin L2 learners of English used an answer sheet indicating if each heard trial was composed of two identical or two different rhythmic phrases. The remaining participants performed the experiment via the computer and their responses were computed by pressing the corresponding “yes-key”, in cases of identical phrases, or the “no-key” in cases of non-identical phrases. The position of the correct-response key (“yes-key”) was counter-balanced across participants.

#### Statistical Analysis

For the purpose of the current work, only language skills involving the explicit (i.e., speaking and listening) or implicit (i.e., reading) use of rhythm (cf., Fodor, 2002; Kentner, 2012) were taken into account. In order to compare L2 learners’ L2 listening, reading and speaking skills, three separate Kruskal-Wallis tests were computed, using each skill as dependent variable and *group* (Mandarin, Turkish and Dutch) as a between-subjects factor.

Participants were also compared in terms of their daily exposure to music (number of hours) and years of formal musical training by means of two Kruskal-Wallis tests using group as a between-subjects factor. No statistically significant differences across groups were found regarding participants’ daily exposure to music and formal musical training, *p*s > 0.1, hence, these two variables were no longer pursued.

Additionally, three analyses of variance (ANOVAs) were computed using participants’ mean scores in the melodic aptitude test and in the two conducted cognitive tests as dependent variables and group as a between-subjects factor. Finally, participants’ mean scores in the rhythmic aptitude test were entered in an analysis of covariance (ANCOVA) as a dependent variable with *group* (Mandarin, Turkish and Dutch late L2 learners of English and Turkish monolinguals) as a between-subjects factor. Participants’ scores in each cognitive test, i.e., the Mottier test and the backward digit span, as well as their mean scores in the melodic aptitude test, were entered in the statistical model as covariates.

## Results

### L2 Skills

Participants’ self-reported L2 listening, speaking and reading skills are shown in Table 1.

**Table 1:** **Participants’ self-reported L2 listening, speaking and reading skills**.

	Mandarin late learners of English		Turkish late learners of English		Dutch late learners of English	
Language skill %	*Mean*	*SD*	*Mean*	*SD*	*Mean*	*SD*
L2 Listening	80.66	10.99	88.00	12.64	91.33	10.60
L2 Speaking	78.00	11.46	85.33	15.05	85.33	15.52
L2 Reading	85.33	10.60	88.00	9.41	91.33	9.90

**Figure 1:**
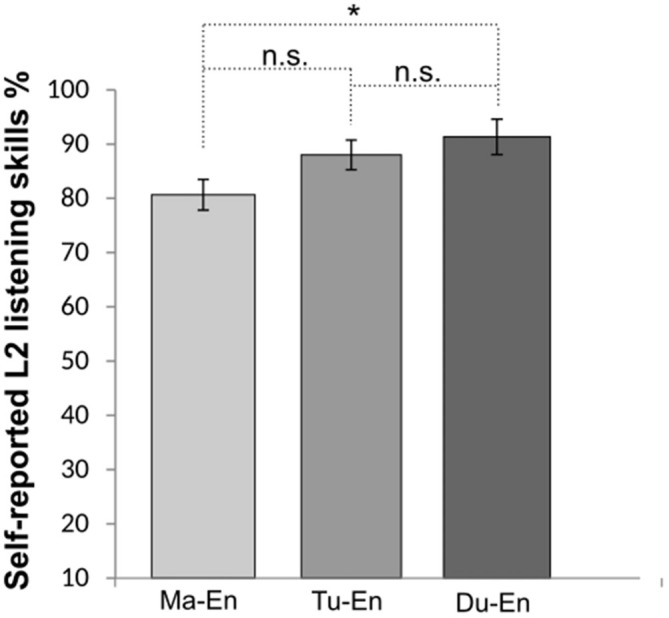
**Self-reported second language (L2) listening skills for Mandarin (Ma-En), Turkish (Tu-En) and Dutch (Du-En) L2 learners of English.** Error bars indicate standard errors. *Statistical significance with *p* < 0.05.

Regarding participants’ L2 (English) skills^5^, no statistical differences were found among groups for L2 reading and speaking skills, *p*s > 0.1, hence these two variables were no further pursued. A significant group difference was found for participants’ L2 listening skills, *X^2^* = 7.23, *p =* 0.02, *r* = 0.93. Pairwise comparisons of the group means using Bonferroni correction revealed a significant difference between Mandarin (*M* = 80.66%, *SD* = 10.99) and Dutch L2 learners of English (*M* = 91.33%, *SD* = 10.60), *p* < 0.016. No statistically significant difference was found between Mandarin (*M* = 80.66%, *SD* = 10.99) and Turkish (*M* = 88.00%, *SD* = 12.64) and between Turkish and Dutch L2 learners of English (*M* = 91.33%, *SD* = 10.60), *p >* 0.016. The mean comparisons of participants’ self-reported L2 listening skill are illustrated in Figure 1.

### Mottier Test, Backward Digit Span, Melodic and Rhythmic Aptitude Tests

Participants’ scores in the Mottier test, backward digit span, melodic and rhythmic aptitude tests, years of formal musical training participants received and daily exposure to music (hours) are depicted in Table 2.

**Table 2:** **Participants’ scores in the Mottier test, backward digit span, melodic and rhythmic aptitude tests, formal musical training and daily exposure to music**.

	Mandarin L2 learners of English		Turkish L2 learners of English		Dutch L2 learners of English		Turkish monolinguals	
Tasks	*Mean*	*SD*	*Mean*	*SD*	*Mean*	*SD*	*Mean*	*SD*
Mottier test	25.53	3.79	24.73	6.09	27.40	4.04	23.40	4.30
backward digit span	10.26	2.84	7.53	2.55	8.00	2.26	6.60	2.09
Melodic aptitude test (%)	76.66	6.24	73.07	9.50	68.07	12.54	53.97	9.89
Rhythmic aptitude test (%)	75.64	6.15	73.97	7.11	66.15	8.77	54.35	10.31
Formal musical training (years)	1.86	2.77	2.46	3.13	1.40	1.05	1.88	1.44
Daily exposure to music (hours)	2.11	1.99	2.70	1.93	1.42	1.06	1.88	1.44

#### Mottier Test and Backward Digit Span

Results revealed no group differences in participants’ scores in the Mottier test, *p* = 0.13. Analysis of participants’ backward digit span score showed a significant group difference, *F*_(3,56)_ = 6.02, *p* = 0.001, *η*^2^ = 0.24. *Post hoc* Boferroni comparison of groups’ mean scores revealed that Mandarin L2 learners outperformed the other groups. No further group differences were found.

#### Melodic Aptitude Test

**Figure 2:**
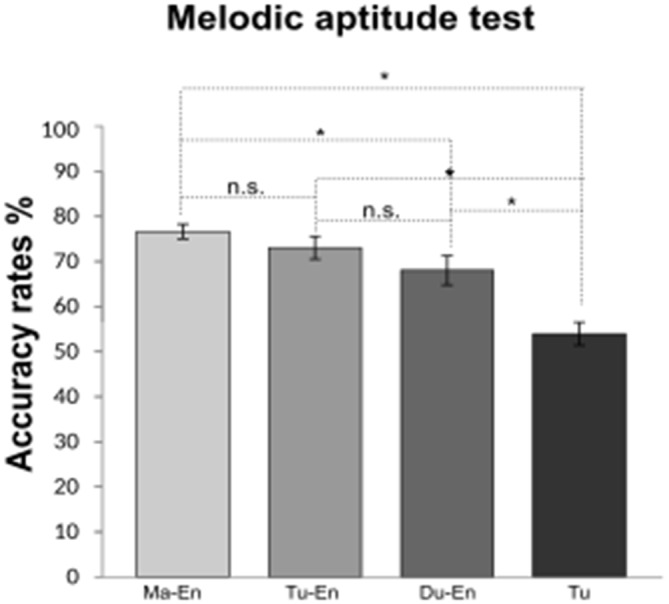
**Accuracy rates in the melodic aptitude test of Mandarin (Ma-En), Turkish (Tu-En), Dutch (Du-En) L2 learners of English and Turkish monolinguals (Tu).** Error bars indicate standard errors. *Statistical significance with *p* < 0.05.

The analysis of participants’ mean scores in the melodic aptitude test revealed a significant effect of group, *F*_(3,56)_ = 15.47, *p* < 0.001, *η*^2^ = 0.12. *Post hoc* comparisons using Bonferroni test revealed a significant difference between monolinguals and L2 groups, with worse performance found for monolinguals (*M* = 53.97%, *SD* = 9.89) than L2 learners (*M* = 72.60%, *SD* = 10.19). A marginally significant group difference was found for L2 learners groups, *p* = 0.06, *η*^2^ = 0.05. Planned comparisons of groups’ mean scores using Bonferroni correction for multiple comparisons revealed higher melodic mean scores for Mandarin (*M* = 76.66%, *SD* = 6.24) than for Dutch L2 learners of English (*M* = 68.07%, *SD* = 12.54). No statistically significant difference was found between Mandarin and Turkish L2 learners (*M* = 73.07%, *SD* = 9.50) and between Turkish and Dutch L2 learners of English. Comparisons of participants’ accuracy rates in the melodic aptitude test are depicted in Figure 2.

#### Rhythmic Aptitude Test

**Figure 3:**
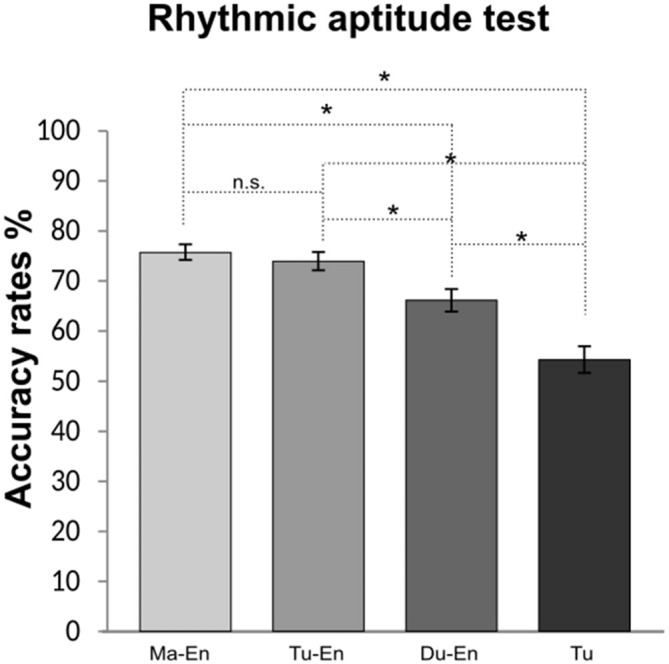
**Accuracy rates in the rhythmic aptitude test of Mandarin (Ma-En), Turkish (Tu-En), Dutch (Du-En) L2 learners of English and Turkish monolinguals (Tu).** Error bars indicate standard errors. *Statistical significance with *p* < 0.05.

For participants’ rhythmic aptitude test, the conducted ANCOVA revealed a significant effect of *group*, *F*_(5,54)_ = 16.31, *p* < 0.001, *η*^2^ = 0.39. A *post hoc* pairwise comparison of participants’ mean scores using a Bonferroni test revealed a significant difference between the Mandarin (*M* = 75.64%, *SD* = 6.15) and the Dutch L2 learners of English (*M* = 66.15%, *SD* = 8.77); and between the Mandarin L2 learners and the Turkish monolinguals (*M* = 54.35%, *SD* = 10.31). Similarly, a statistically significant difference in mean scores was found between the Turkish (*M* = 73.97%, *SD* = 7.11) and the Dutch L2 learners of English (*M* = 66.15%, *SD* = 8.77) and between Turkish L2 learners and monolinguals (*M* = 54.35%, *SD* = 10.31). A significant group difference in mean scores was also encountered when comparing Turkish monolinguals with Dutch L2 learners of English. Hence, Turkish monolinguals performed worse than all the other groups. No statistically significant difference was found between Mandarin and Turkish L2 learners groups. Comparisons of participants’ accuracy rates in the rhythmic aptitude test are depicted in Figure 3.

To investigate if rhythmic performance in the three L2 learners group could be affected by the difference in their L2 listening skill, an additional ANCOVA was computed adding L2 listening skill to the other covariates, i.e., participants’ scores in the Mottier test, backward digit span and melodic aptitude test. Results revealed that L2 listening skill does not contribute significantly to participants’ rhythmic performance, *F*_(8,36)_ = 0.69, *p* = 0.42. Therefore, this variable was not further pursued.

## Discussion

The current research investigated whether and how the learning of a L2 could contribute to individuals’ musical rhythmic perception. Turkish monolinguals and three groups of L2 learners, namely, Mandarin, Turkish and Dutch L2 learners of English were tested on their rhythmic perception in music. Additionally, Turkish monolinguals were tested to account for the possibility that the exposure to musical rhythmic complexity could explain a possible enhancement in individuals’ musical rhythmic perception. To account for individual differences in cognitive ability and musical aptitude, which could have influenced participants’ rhythmic perception, participants’ working memory, phonological memory and melodic aptitude were assessed, and used as covariates.

Our results showed L2 learning to be more salient to musical rhythmic perception than exposure to musical rhythm complexity, since Turkish monolinguals demonstrated worse performance than all the other groups, including Turkish L2 learner of English. Additionally, monolinguals performed worse than L2 learners in the melodic aptitude test, despite that no group differences were found between monolinguals and their L2 learner peers regarding their formal musical training, daily exposure to music and phonological memory. Interestingly enough, the only group difference found with respect to the cognitive measures here collected concerned the higher working memory scores of Mandarin L2 learners of English in comparison with the other groups. According to previous research, differences in verbal working memory could be due to cultural differences (cf., Hedden et al., 2002). Thus, the use of non-verbal working memory measures in future cross-cultural studies could be an option if one wishes to avoid such differences.

The worse performances of Turkish monolinguals in musical rhythm and melody perception in comparison with the three L2 learners groups could indicate that learning an L2 might enhance the overall perception of acoustic variation, such as the variation in sound duration, intensity and pitch. Similarly to how musical training may shape one’s auditory skills (Vuust et al., 2012), learning a L2 could promote similar effect. Furthermore, the enhanced melodic perception of Mandarin in comparison with Dutch L2 learners of English corroborates previous findings in the literature (Wong et al., 2012; Bidelman et al., 2013) that report enhanced musical pitch perception in native speakers of tonal in comparison with those of non-tonal languages. The lack of group difference between the melodic performance of Mandarin and Turkish L2 learners could be due to the size of the effect which, despite a visible trend, failed to reach significance. Therefore, future research further contrasting the melodic aptitude of tonal and non-tonal L2 learners should be carried out.

Regarding L2 learners’ rhythmic performance, our findings indicate that an enhanced rhythmic perception is found for L2 learners whose first and second languages diverge in their rhythmic characteristics, as is the case of Mandarin and Turkish L2 learners of English. Perhaps Dutch L2 learners of English could be worse at musical rhythmic perception than Turkish and Mandarin L2 learners of English due to the full overlap of rhythmic properties between these two languages.

The comparable performances of Mandarin and Turkish L2 learners of English could indicate that the processes of reconfiguring stress position (from word-final to word-initial position) and learning a new lexical stress system could enhance one’s rhythmic perception. This could be the case because having to learn rhythmic features in L2 that are different from the native language, such as sound duration and intensity, could make one more aware of variations in these rhythmic features in language and musical perception.

The observed enhanced rhythmic perception could represent another cognitive advantage of bilinguals, similar to verbal and non-verbal intelligence (Peal and Lambert, 1962), problem solving skills (Bialystok, 1999; Bialystok and Shapero, 2005), and phonological memory (Service, 1992; Cheung, 1996). An enhanced rhythmic perception could be decisive to a more successful language encoding (Sundara and Scutellaro, 2011), language recognition and a more effective selection of the target-language (cf.,Roncaglia-Denissen et al., 2013a).

Rhythmic information is not only relevant for language, but also for music organization. Thus, a perceptual auditory enhancement in language could be transferred and used in the music domain, as our results seem to suggest. Evidence of cognitive transfer between the language and the music domains has been reported by quite a few studies. On the one hand, the use of linguistic pitch variation by tonal native speakers enhances their perception of musical pitch variation (Deutsch et al., 2006; Elmer et al., 2011), and on the other hand, musical training improves the perception of linguistic pitch variation (Slevc and Miyake, 2006; Marques et al., 2007; Milovanov et al., 2008). Regarding rhythmic skills, it has been shown that effects of rhythmic training can be transferred to the language domain (Bhide et al., 2013), and timing sensitivity in language may be predicted by musical aptitude (Milovanov et al., 2009; Marie et al., 2011; Sadakata and Sekiyama, 2011). The present study is in line with previous research, suggesting that learning languages with distinct rhythmic properties enhances individuals’ perception of rhythmic variation in music (Roncaglia-Denissen et al., 2013a; Bhatara et al., 2015). The existence of a bi-directional transfer effect between the language and the music domain strongly suggests the existence of shared mechanisms and cognitive resources between them (Patel, 2008, 2014).

In face of the reported results one may argue that, together with learning an L2, other unmeasured cultural variables could be contributing to our findings. If this should be the case, future investigations should address this matter. Additionally, a few concrete questions remain, such as which rhythmic features of speech might contribute to the enhancement of individuals’ musical rhythmic perception. The processing of timing features in music has been described as having different levels, from the encoding of short timing span (Repp, 2005) to an overall rhythmic pattern analysis of longer sound sequences (Zanto et al., 2006).

In speech, timing information provides important cues at different levels as well. At the phonological level, it helps to distinguish vowels, e.g., in Dutch, (Booij, 1999) and consonants, e.g., in Japanese (Han, 1992; Sadakata and McQueen, 2013; Kawahara, 2015). At the word level, timing information manifests itself as word metric preference, e.g., the trochee or the iamb (Hayes, 1985), while beyond the word level, it helps to organize the speech flow (Grabe and Low, 2002; Roncaglia-Denissen et al., 2013b). The sensitivity to such timing cues depend on one’s mastered languages (Kingston et al., 2009; Sadakata and Sekiyama, 2011; Roncaglia-Denissen et al., 2015).

Perhaps mastering languages with distinct word metric preference, e.g., word initial vs. word-final stress, may be enough to enhance rhythmic sensitivity in music. Alternatively, perhaps being sensitive to broader features such as speech organization units, e.g., metric foot or the syllable, as for our Mandarin learners of English, may be enough to enhance rhythmic sensitivity. It could also be that the interplay between the word and speech levels, rather than their respective impact alone, account for such a rhythmic enhancement.

To disentangle which mechanisms are playing a central role in enhancing individuals’ rhythmic perception, be it word metric preference, speech organization, or both, one could extend the current approach to other language pairs that diverge in their rhythmic features. For instance, a language pair consisting of a syllable-timed language and a stress-timed language that share the word metric preference for the trochee, as it is the case of Spanish and English respectively (Pike, 1945; Jusczyk et al., 1993; Sebastian and Costa, 1997; Schmidt-Kassow et al., 2011), would be a good candidate for investigation. Additionally, two syllable-timed languages with different metric preference, such as Turkish (with the preference for word-final stress) and Spanish (a word-initial language) would also prove an interesting investigation. Future research addressing this matter will help us truly understand which relevant features for rhythmic perception in language could be also relevant and could be used in musical rhythmic perception. With this knowledge, one could gain a better understanding of what the music and language domains might share, and be one step closer to grasping what makes these two domains so unique and particular to humans.

## Author Contributions

MPRD, DAR, AC and MS contributed to the design of the experiment, to the recruitment of participants and data collection. MPRD was responsible for data analysis. MPRD, DAR, AC and MS contributed to the writing of the manuscript. MPRD was the lead author. DAR, AC and MS contributed to different sections of the manuscript and reviewed drafts of it.

## Conflict of Interest Statement

The authors declare that the research was conducted in the absence of any commercial or financial relationships that could be construed as a potential conflict of interest.

## Footnotes

^1^The use of rhythmic categories, such as syllable-timing, stress-timing and mora-timing, has been challenged by some of the field literature (e.g., Grabe and Low, 2002; Nolan and Asu, 2009) suggesting that a rhythmic continuum, instead of categories, would be a more adequate characterization of language rhythms. This would be the case because one language could present characteristics of both categories.

^2^A priori power analysis was conducted (GPOWER; Erdfelder et al., 1996) based on the large effect size (ω^2^ = 0.32) of the relationship between musical rhythmic ability and language group reported by Roncaglia-Denissen et al. (2013a). This power analysis indicated that a sample size of 42 L2 learners (13 participants per group) would be sufficient to detect group differences with a power of 0.95 and an alpha of 0.05. To keep groups’ size comparable, the sample size of monolingual participants was also estimated in 13 participants.

^3^The younger age of Turkish monolinguals in comparison with the other participants is due to the fact that they were in their first semester as university students at time of data collection. The monolingual participants had been already accepted as university students, but were taking English basic course to pass an English proficiency exam required to continue their education at the Middle East Technical University, in Ankara.

^4^Mandarin L2 learners of English were tested in Utrecht, Tilburg and in Amsterdam. Turkish L2 learners of English were tested in Amsterdam, Tilburg, Nijmegen and in Ankara. Dutch L2 learners of English were tested in Amsterdam, while Turkish monolinguals were tested in Ankara.

^5^In addition to the self-reported L2 language (English), Turkish participants reported having very basic knowledge of Chinese (1 participant), German (5 participants), French (2 participants), Japanese (1 participant), Finish (1 participant), intermediate knowledge of French and Dutch (1 participant) and advanced knowledge of Dutch (1 participant). Chinese participants reported having very basic knowledge of Korean (2 participants), Japanese (4 participants), Russian (1 participant), French (1 participant) and Dutch (5 participant). Dutch participants reported having very basic knowledge of French (4 participants), Spanish (3 participants), Czech (1 participant) and an intermediate knowledge of German (10 participants).
