# Supplementary material for: The Enhanced Musical Rhythmic Perception in Second Language Learners
Source: Front Hum Neurosci. 2016 Jun 10;10:288. doi: 10.3389/fnhum.2016.00288 (PMC4901070; doi:10.3389/fnhum.2016.00288)
Supplement: Supplementary file 1 [file DataSheet_1.pdf]

Music background Questionnaire

1a) Name: ____

1b) Birthdate: ____

1c) Gender: Feminine ☐ Masculine ☐ Other ☐

2a) Have you had any formal music lessons in school, or have you done any courses in general music theory?

Yes ☐

No (go to question 3) ☐

2b) If yes, how old were you when you started these lessons?

2c) At what age did you stop having these lessons?

2d) Notes/Remarks:
Please add any remarks about the questions 2a), 2b), 2c) you think are necessary.

3a) Have you followed dance lessons?

Yes ☐

No (go to question 4) ☐

3b) If yes, how many years of lessons have you had?

1-2 years ☐

3-5 years ☐

More than 5 years ☐

Other ☐

4a) On average, how many hours per day do you listen to music?

___

4b) Per genre, can you specify in percentages which to music genres you listen to? The sum of all percentages should be 100%.

Please choose from the following genres or add your own genres, if you think it is necessary: Classical Music, Pop music, Jazz music, Rock music, World/Folk music.

5a) Do you go to concerts?

Yes ☐

No (go to question 6) ☐

5b) If yes, how often?

Weekly ☐

1-2 per month ☐

2-10 times per year ☐

Less than 2 times per year ☐

5c) If yes, which genre(s)?

Please add any extra information you think is necessary.
